# Isolated Non-Progressive Hemidystonia in a Patient Homozygous for H63D Variant of Hereditary Hemochromatosis: A Case Report and Systematic Literature Review of Movement Disorders in Hereditary Hemochromatosis

**DOI:** 10.3390/diagnostics15172275

**Published:** 2025-09-08

**Authors:** Stefania Kalampokini, Andreas Plaitakis, Cleanthe Spanaki, Georgia Xiromerisiou

**Affiliations:** 1 First Department of Neurology, Aristotle University of Thessaloniki, AHEPA University Hospital, Stilponos Kyriakidi 1, 54636 Thessaloniki, Greece; 2 Department of Neurology, School of Health Sciences, Faculty of Medicine, University of Crete, Voutes, 71003 Heraklion, Greece; andreasplaitakis@gmail.com (A.P.); kliospanaki@gmail.com (C.S.); 3 Institute of Preventive Neurology and Brain Health, Mindful Mind, Aigaiou 45, 55133 Thessaloniki, Greece; georgiaxiromerisiou@gmail.com

**Keywords:** hereditary hemochromatosis, H63D mutation, dystonia, movement disorders

## Abstract

**Background**: Hereditary hemochromatosis (HH) is a genetic disorder of iron metabolism, characterized by progressive iron accumulation. Neurological involvement, which can manifest with various symptoms, including movement disorders, is uncommon. **Methods**: We describe a case of a 50-year-old male patient homozygous for the H63D variant of the HFE gene (encoding the human homeostatic iron regulator protein), who also carried the c.340+4T>C polymorphism in the same gene and has been affected since the age of 13 years by hemidystonia involving primarily his right upper extremity. His brain MRI, obtained approximately 35 years after initial symptoms, revealed iron deposition predominantly in the contralateral pallidum. The patient has shown no progression of his neurologic syndrome and no systemic manifestations over the 35 years of follow-up. Moreover, we conducted a comprehensive literature search in Pubmed and Web of Science in English of all previously reported cases of movement disorders due to HH. **Results**: We found 19 studies including 69 patients with movement disorders. Movement disorders associated with HH were, in most cases, hypokinetic and less commonly hyperkinetic. The most common movement disorders were tremor, parkinsonism, ataxia, and less frequent dystonia, chorea, and myoclonus. Movement disorders could either precede the diagnosis of HH, or they could occur with a variable latency ranging from a few months up to 12 years after disease onset. Iron deposition on brain MRI in the basal ganglia or cerebellum was found in few of those cases. **Conclusions**: The association between hemochromatosis and movement disorders is rare. Blood analysis, including serum iron, ferritin, and transferrin saturation levels, should be investigated in patients with movement disorders of unknown etiology or with iron deposition on neuroimaging. A better understanding of genotype-phenotype correlations would facilitate the early diagnosis of HH.

## 1. Introduction

Hereditary hemochromatosis (HH) is a genetic disorder of iron metabolism, characterized by progressive iron accumulation leading to functional impairment of various organs [1]. HH is commonly caused by mutations in the HFE gene, located on 6p22.2, which encodes the human homeostatic iron regulator protein (HFE protein), a transmembrane protein of 321 amino acids, which acts as a modulator of cellular iron uptake [1]. HFE is expressed not only in intestinal and immune cells, but also in neurons and glia [2]. More than 30 different mutations in the HFE gene have been implicated in HH; the most frequent being C282Y, H63D, and S65C [3]. Individuals with HH commonly harbor the homozygous C282Y mutation, leading to excessive iron absorption, with a phenotypic penetrance of less than 25% [4]. On the other hand, the prevalence of the heterozygous HFE H63D is 21% and the homozygous state 1% [3].

Clinical manifestations of HH span a broad spectrum, including chronic fatigue, liver complications, cardiomyopathy, arthritis, diabetes mellitus, and bronze skin pigmentation [5]. Serum ferritin levels are commonly increased, i.e., above 300 ng/mL in men and 200 ng/mL in women, along with serum transferrin-iron saturation exceeding 45% [5]. Neurological involvement is uncommon, but when present, it may manifest as lethargy, cognitive decline, depression, or gait difficulties [3,6]. Movement disorders are rare, with only a few reported cases [6,7,8,9,10,11,12,13]. While H63D has a higher prevalence than C282Y mutation, neurologic involvement is exceedingly rare in H63D homozygotes [14]. Here, we describe a case of a patient carrying a homozygous H63D mutation presenting with hemidystonia at puberty, without any other systemic manifestation of HH, who was followed up for over three decades. Moreover, we conducted a detailed literature search regarding all reported cases of hypokinetic or hyperkinetic movement disorders that have been associated with HH.

## 2. Case Presentation

The patient has been personally evaluated and followed longitudinally for more than 35 years by one of the authors (AP). The case is a 50-year-old male patient who was in a usual state of health until the age of 13 years, when, during a school excursion, he experienced constitutional symptoms associated with transient episodes of involuntary head rotation to the left. Speech disturbances, sialorrhea, dysphagia to liquids, and difficulty using the right hand soon followed. Within days, involuntary contractions of the flexor muscles of the right forearm and right fingers developed, resulting in markedly abnormal posturing of the right upper extremity and inability to use the right hand. The symptoms were persistent, although with some degree of fluctuation. They were alleviated during sleep and were less pronounced after awakening. His past medical history was unremarkable, with normal birth and neurodevelopmental milestones. There was no family history of a similar neurological disorder.

Initial neurological examination in the summer of 1988 (six months after the initial symptoms) at the age of 13 years, revealed dysarthria, slight sialorrhea, and hemidystonia affecting predominantly the right upper extremity. The right arm was adducted at the shoulder, forcefully flexed, and pronated at the elbow. The right wrist was dorsally extended, and the right fingers were flexed, showing athetoid movements. The right leg showed circumduction on walking, but showed no postural abnormalities at rest. No sensory abnormalities and no pyramidal involvement were found. Also, no clinical signs of parkinsonism were detected. A laboratory work-up, including blood counts, erythrocyte sedimentation rate, routine blood chemistries, thyroid studies, serum cortisol levels, anti-nuclear antibodies, cerebrospinal fluid analysis (cell count, antibodies against toxoplasmosis, Cytomegalovirus, Rubella, Measles and Poliovirus) were unremarkable. Slit lamp cornea examination revealed no abnormalities, while serum coeruloplasmin was increased (51 mg/dL) with normal urine copper levels. Magnetic Resonance Imaging (MRI), obtained in 1988, was reported to be normal, but the study has not become available to us. Nerve conduction studies, and electroencephalogram, performed at that time, were unremarkable.

The patient was initially treated with carbamazepine (up to 200 mg QID) without clinical improvement. He subsequently received levodopa/carbidopa 100/10 up to four times daily, with additional carbidopa, in order to enhance inhibition of the peripheral L-amino acid decarboxylate. On this regimen, no substantial clinical response was observed. Then, treatment with a gradually increasing dose of trihexyphenidyl, which is thought to be effective in children with dystonia [15], was initiated. The trihexyphenidyl dosage was carefully increased over eight months to 60 mg/daily, with slight improvement of symptoms. Despite good tolerance, the clinical response was not considered substantial to justify continuation of trihexyphenidyl, which was slowly tapered and eventually discontinued. By the time the dose had been reduced below 17 mg/daily, the patient experienced an episode of oculogyric crises during which his eyes turned upwards, associated with anxiety and increased dystonic phenomena. A similar episode of oculogyric crises had also occurred shortly after the onset of symptoms. In 1994 (at the age of 19 years) the patient was started on clonazepam 0.5 mg, taken orally TID. On this treatment, he experienced mild clinical improvement. Treatment with Botulinum toxin was also attempted, but proved no clear benefit and, as such, it was not further pursued.

Approximately 35 years later, the patient was re-evaluated at the age of 50. Neurological examination revealed a rather stable movement disorder characterized by right-sided hemidystonia involving predominantly the right upper extremity. No fixed deficits, such as muscle contractures or joint ankyloses, had developed. When prompted, the patient was able with great effort to temporarily extend his right arm at the elbow and to open his right fingers, which showed some athetoid movements (Video S1). Under these conditions, the muscle power of the right extremity was normal. The deep tendon reflexes were also normal, and the plantar response was flexor. The sensory examination was normal bilaterally. The left extremities showed normal muscle tone and power and no reflex abnormalities. There was no tremor or cogwheeling rigidity. Moreover, no other signs of parkinsonism were observed. Bulbar dysfunction was no longer present. In summary, the patient’s neurological status has remained essentially unchanged, and the mild clinical improvement on clonazepam has been sustained over the years.

**Figure 1:**
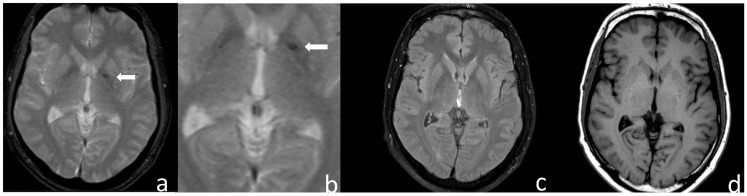
Patient’s brain MRI. (**a**) Susceptibility-weighted imaging (SWI) sequence revealing low signal intensity in the left pallidum (white arrow), with lesser involvement of the right pallidum, consistent with iron deposition on the basal ganglia. (**b**) Magnified SWI image, (**c**) normal T2-weighted signal, and (**d**) normal T1 signal.

A brain MRI, obtained at the age of 50, revealed low signal intensity in the left pallidum, with lesser involvement of the right pallidum in susceptibility-weighted imaging (SWI), indicative of iron deposition in the basal ganglia (Figure 1). T2- and T1-weighted MRI sequences showed normal signal. Computer tomography of the brain excluded basal ganglia calcifications. Subsequent laboratory investigations revealed significantly elevated iron parameters: markedly elevated serum ferritin (1158 mg/mL), elevated serum transferrin saturation (>65%), and gGT (119 U/L).

Genetic analysis confirmed a homozygous mutation in exon 2 of the HFE gene (NM_000410.4:c.187C>G, p.(His63Asp)). This mutation results in the substitution of histidine with aspartate at position 63 in the HFE protein. Histidine is located within a functionally significant region of the protein [16], and its replacement with aspartate represents a substantial physicochemical change (Grantham score: 81) [17]. This mutation is classified as pathogenic in ClinVar [18], supporting its role in the patient’s condition. The patient also had the polymorphism c.340+4T>C in HFE gene. This variance is found in an area of the protein without known functional significance and does not affect the protein reading frame [19,20].

Given these findings, further imaging was performed to assess potential systemic iron deposition. A T2*-weighted MRI of the heart and liver was conducted to exclude cardiac and hepatic iron overload. The results indicated the absence of pathological iron accumulation in both heart and liver and normal ventricular size and function, indicating no cardiac dysfunction secondary to iron overload.

## 3. Materials and Methods

**Figure 2:**
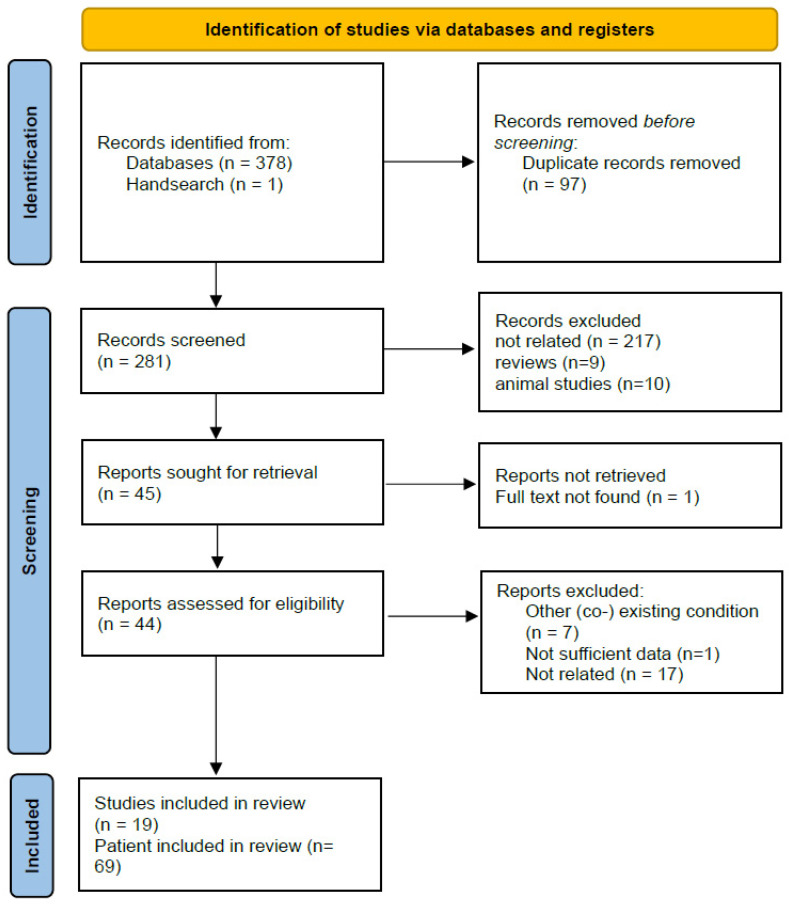
Flowchart of the included studies.

We conducted a systematic literature review in databases MEDLINE and Web of science in English in all records till May 2025. The keywords used were “hereditary hemochromatosis OR hemochromatosis OR haemochromatosis”, “parkinsonism OR parkinson’s disease”, “extrapyramidal”, “tremor”, “ataxia”, “dystonia”, “chorea”, “myoclonus OR jerky”, “tics”, “hypokinetic OR hyperkinetic movement disorders”. Inclusion criteria were English language, original studies/case reports or series. Exclusion criteria were articles not written in English, articles without abstract, articles that involved laboratory animals, and non-original studies (reviews, editorials, expert opinions). No better explanation for the presence of movement disorders should be present, i.e., papers with co-existing conditions that could cause movement disorders were excluded. Reference lists of the retrieved records were checked for additional potentially eligible studies. From each eligible paper, the information extracted was as follows: number of patients, gender, age at presentation, mutation, movement disorders, other neurological symptoms, other systemic symptoms or features, iron deposition on brain MRI, treatment, outcome on movement disorder. In total, 19 studies reporting cases of 69 patients were included in the systematic review. The flow chart of the included studies can be seen in Figure 2.

## 4. Discussion

The association between hemochromatosis and movement disorders is rare. The previously described cases of movement disorders in patients with HH, can be seen in Table 1. Here, we report the first case of a patient homozygous for the H63D variant presenting with non-progressive hemidystonia with subacute onset and MRI-confirmed deposition of iron in the contralateral pallidum, without other systemic manifestations of HH. Demarquay et al. reported a case of a 35-year-old man with a homozygous C282Y mutation, who presented with mild cervical dystonia, head and arm tremor, cerebellar ataxia, dysarthria, and hypotonia [7]. His brain MRI revealed cerebellar atrophy without basal ganglia or white matter changes [7]. Similarly, Sharma et al. reported three patients with dystonic tremor, two of whom had bilateral symptoms [21]. Increased susceptibility in the thalamus was found on MRI, particularly contralateral to the limb with dystonic tremor [21]. Ragunatham et al. reported the case of a female patient with HH presenting with long-standing orofacial and cervical dystonia and hemosiderin deposition in the basal ganglia and dentate nuclei [22]. Lastly, Russo et al. reported the case of a 63-year-old woman with progressive gait disturbance, chorea, and mild cervical and laryngeal dystonia, who was diagnosed with HH and Huntington’s chorea [23].

Other movement disorders previously described in HH comprise parkinsonism [6,7,9,10,13,24,25,26], cerebellar ataxia [7,8,9,11,12], tremor [7,9,10,13,24], myoclonus [7,8], chorea [24] and restless legs syndrome [25,27,28]. Movement disorders due to HH were, in most cases, hypokinetic and less commonly hyperkinetic. The most common movement disorders were tremor, usually rest and/or postural, parkinsonism and ataxia, while less frequent were dystonia, chorea or myoclonus. They may precede the diagnosis of HH [7,9,11,13,25,26,27] or appear after a variable latency ranging from a few months to 12 years after disease onset [6,8,12,23,24,28]. Parkinsonism could be bilateral or unilateral, mimicking idiopathic Parkinson’s disease. In two reported cases, dopamine transporter (DAT) scan revealed reduced uptake in the basal ganglia, either unilaterally [25] or bilaterally [29]. A large prospective cohort study showed that male p.C282Y homozygote patients had a higher cumulative incidence of Parkinson’s disease at the age of 80 years compared to individuals without these pathogenic variants [30]. However, two meta-analyses found no association of C282Y and H63D polymorphisms in hemochromatosis gene and risk of Parkinson’s Disease [31,32]. Ataxia due to HH can present as either limb or gait ataxia, with or without dysarthria [7,8,9,11,12,23], while nystagmus in ataxia due to HH has not been reported. There has been one case with co-existing Friedreich ataxia and hemochromatosis, which had a more severe phenotype than Friedreich ataxia alone [33]. Another study showed that p.C282Y heterozygosity is associated with an earlier age at onset of Friedreich’s ataxia, probably related to exacerbation of the already dysregulated iron metabolism that plays a major role in the pathogenesis of Friedreich’s [34]. Chorea was rarely reported, and it was asymmetrical [24]; in fact, in two cases, there was co-existence with Huntington’s disease, which may imply a synergistic genetic effect [23,35]. Other neurological symptoms or signs were cognitive decline, postural hypotension and brisk reflexes. Similar to the present case, there were also few cases of HH presenting with predominant neurological features without any or minimal systemic findings [6,7,13,21,24,27,28], which led Kumar et al. to propose the term “neuro-hemochromatosis” [24]. The most common non-neurological symptoms or signs were diabetes mellitus, skin pigmentation, increased ferritin and transferrin saturation, and less commonly hepatomegaly.

**Table 1:** Reported cases with hemochromatosis and movement disorders.

Authors, Year	Gender	Age at Presentation	Mutation	Movement Disorders	Other Neurological Symptoms	Presenting Symptom as Movement Disorder	Other Systemic Features	MRI Brain/Iron Deposition	Treatment	Outcome on Movement Disorder
Jones and Hedley-Whyte, 1983 [8] Case 1	Male	71	n/a	Ataxia, rigidity, myoclonic jerks	dementia	no	Diabetes, hemochromatosis in liver biopsy	Basal ganglia calcification on CT	40 gr protein/d, neomycin	died due to hepatic encephalopathy a few weeks later
Jones and Hedley-Whyte, 1983 [8] Case 2	Male	52	n/a	Ataxia	Lethargy, dysarthria	no	Weight loss, skin pigmentation, hepatomegaly	n/a	Phlebotomies	Initial improvement, died after few months
Nielsen, 1995 [9]	Male	50	HLA-A2,3, B7, 44 heterozygosity	Left-sided parkinsonism, ataxia, postural and rest tremor	cognitive problems, headache, pyramidal weakness	yes	Fatigue, bronze skin pigmentation, heavy iron overload in liver, high ferritin and transferrin saturation	Reduced T2 signal/iron accumulation in the caudate, globus pallidus, red nucleus, substantia nigra, dentate nucleus	Levodopa Phlebotomies	Improvement of parkinsonism with levodopa
Demarquay et al., 2000 [7]	Male	35	Homozygous C282Y	Postural head and arm tremor, ataxia, cervical dystonia	Dysarthria	yes	Bronze skin pigmentation, heavy iron accumulation in liver biopsy	Moderate cerebellar atrophy	Phlebotomies, clonazepam, buspirone, trihexyphenidyl	No improvement with phlebotomy, little benefit of medication
Costello, 2004 [6] Case 1	Male	55	Homozygous C282Y	resting tremor, parkinsonim	no	no	DM, elevated ferritin	n/a	Levodopa Phlebotomies	Good response to levodopa, no effect of phlebotomy
Costello, 2004 [6] Case 2	Male	61	HLA A2A3B7B12 haplotype	Bilateral parkinsonism	no	no	DM II, abnormal skin pigmentation, elevated ferritin and transferrin saturation, cirrhosis on liver biopsy	Atrophy (not specified) on CT	Levodopa	Good response on levodopa
Costello, 2004 [6] Case 3	Male	63	Homozygous C282Y	Lower limb and body tremor, bilateral parkinsonism	no	yes	Abnormal liver function tests, siderosis on liver biopsy, elevated ferritin	Normal brain MRI	Levodopa	Good response
Costello, 2004 [6] Case 4	Female	49	Homozygous C282Y	Asymmetrical rest tremor and parkinsonism	no	no	DM II, elevated ferritin, heavy iron deposition on liver biopsy, angina	Cerebral infarcts on CT	Phlebotomies, levodopa	Iron overload corrected with phlebotomy/ Good response to levodopa
Dethy, 2004 [27]	Female	62	C282Y mutation	Restless legs, arm tremor, parkinsonism	no	yes	Increased ferritin, transferrin saturation, iron deposition on liver MRI	Normal brain MRI	Phlebotomies	After five phlebotomies neurological signs subsided
Haba-Rubio et al. 2004 [28] Case 1	Female	40	Homozygous C282Y	Restless legs	no	yes	no	Reduced R2’ values (reduced iron level) in substantia nigra, red nucleus, pallidum	Phlebotomies, clonazepam	No improvement with phlebotomies, improvement with clonazepam
Haba-Rubio et al. 2004 [28] Case 2	Male	56	Homozygous C282Y	Restless legs	Apathy, fatigue, lack of libido	no	Iron overload in liver biopsy	Reduced R2’ values (reduced iron level) in the substantia nigra, red nucleus, pallidum	Phlebotomies	Deterioration
Shaughnessy et al. 2005 [36] 10 patients	7 Male	53 (41–76)	Mainly C282Y homozygous	Restless legs	n/a	Yes (5 out of 10)	Low level ferritin, no diabetes, rest n/a	n/a	Phlebotomies	one improved, three worsened, one no significant change, rest n/a
Rutgers, 2007 [11]	Female	72	Homozygous C282Y	Ataxia	no	yes	Bronze skin pigmentation, hepatic siderosis on abdominal CT	Decreased signal in putamen and dentate nuclei on GRE	Phlebotomies	stable
Rosana, 2007 [10]	Male	60	Homozygous C282Y	Limb tremor, bilateral parkinsonism	no	no	Elevated ferritin and transferrin saturation, iron accumulation on liver biopsy, arthritis, skin hyperpigmentation	Bilateral globus pallidum and putamen (increased T1, low T2 signal)	Phlebotomies Medication (Levodopa, selegiline, paroxetine, clonazepam, mirtazapine, alprazolam) DBS of right ventral intermediate nucleus	no significant effect No effect of medication Marked improvement after DBS
Di Filippo, 2010 [12]	Male	44	Homozygous H63D	Ataxia	Dysautonomia, brisk reflexes	yes	Elevated ferritin levels, mild hepatomegaly, liver iron deposition	Olivopontocerebellar atrophy, “hot cross bun” sign in pons, no iron deposition on brain MRI	n/a	n/a
Chang et al. 2011 [29]	Female	49	n/a	Bilateral parkinsonism	no	yes	Skin hyperpigmentation, arthropathy, increased serum iron and ferritin	Decreased signal on T2* in bilateral globus pallidus, striatum, thalamus, substantia nigra and dentate nucleus	Chelation therapy (deferoxamine) Levodopa	Partial response to levodopa
Williams, 2013 [13]	Female	60	Homozygous H63D	Right-sided rest tremor/parkinsonism	Postural hypotension, daytime somnolence	yes	Mildly raised ALT	Normal MRI	Dopamine agonists, levodopa	No effect, progression
Ragunathan et al., 2014 [22]	Female	57	Homozygous C282Y	Orofacial and cervical dystonia	Depression, anxiety	yes	Increased ferritn, iron deposition in liver biopsy	Hemosiderin deposition in the basal ganglia and dentate nuclei	Phlebotomies	No improvement
Kumar et al. 2016 [24]	Male	61	Homozygous C282Y	Rest and action tremor of left upper limb, left-sided parkinsonism	no	No	Increased ferritin and transferrin saturation	symmetrical T1 hyperintensity in the lentiform nuclei and substantia nigra bilaterally	Phlebotomies Levodopa-carbidopa	Improvement of parkinsonism with levodopa
Kumar et al. 2016 [24]	Male	64	C282Y homozygous mutation in HFE gene, heterozygous mutation c.212G>A (p.Gly71Asp) in hepcidin gene	Left-sided choreiform movements of the extremities	no	no	Increased serum ferritin and transferrin saturation	T2 hypointensity with low signal on SWI in both caudate, lentiform nuclei, dorsolateral thalami and dentate nuclei consistent with iron deposition	Phlebotomies Tetrabenazine	chorea improved on tetrabenazine
Kumar et al. 2016 [24]	Female	61	Homozygous C282Y	Postural and action tremor	no	no	Increased serum ferritin, transferrin saturation, iron overload in liver biopsy	low signal on SWI in the dentate, red nuclei and substantia nigra	Phlebotomies Propranolol	tremor improved with propranolol
Girotra, 2017 [25]	Male	41	Compound heterozygous (C282Y, H63D)	Bilateral hand rest and action tremor, right-sided parkinsonism, restless legs	Depression, fatigue, anosmia	yes	n/a	Normal MRI	Levodopa	Improvement of tremor
Fatima 2019 [37]	Male	60	Homozygous C282Y	RLS, periodic limb movement disorder (when ferritin dropped to 57 ng/mL after phlebotomy)	n/a	no	Increased ferritin prior phlebotomy (>9000 ng/mL)	n/a	Phlebotomies Dopamine agonists (pramipexole, rotigotine), gabapentin, codeine or combinations	No response to medication Improvement with iron therapy when ferritin dropped
Scarlini et al. 2020 [38]	Male	59	Homozygous C282Y	Right-sided parkinsonism	headache	no	DM II, autoimmune thyroiditis, chondrocalcinosis, liver and heart iron deposition, increased ferritin	T2* hypointensities/calcifications within globus pallidus, substantia nigra, dentate nucleus, left thalamus	Phlebotomies	n/a
Banta, 2023 [26]	Male	55	Compound heterozygous (C282Y, H63D)	Right-sided hand tremor, parkinsonism	no	yes	Skin hyperpigmentation, DM II, arthralgia	Loss of hyperintensity of right substantia nigra on T2	Primidone, pramipexole, ropinirole Phlebotomies	No improvement
Sharma et al., 2021 [21] 35 patients	n/a	61.33 ± 12.66	Most C282Y homozygous	Action tremor, parkinsonism, bilateral dystonic tremor	n/a	no	n/a (no liver involvement)	Increased SWI (iron deposition) in thalamus contralateral to limb with dystonic tremor	Phlebotomies, botulinum toxin, propranolol, primidone, levodopa/carbidopa	n/a excellent response of hand dystonia to botulinum toxin

**Abbreviations:** n/a: not reported, QID: four times per day, DM: diabetes mellitus, GRE: gradient spin echo, DBS: deep brain stimulation, MRI: magnetic resonance imaging, CT: computer tomography, SWI: susceptibility-weighted imaging.

The patients with HH were, in most cases, middle-aged when the movement disorder occurred, with a range of 35–73 years. Apparently, iron accumulation in the brain requires many years before it reaches a certain threshold [39], not to mention the age-related increase in iron content, affecting primarily the globus pallidus, red nucleus, substantia nigra, caudate, thalamus, dentate nucleus and putamen bilaterally [40,41,42,43]. Concerning gender, it seems that movement disorders due to HH occur more often in male than female patients, although the small number of patients with HH and movement disorders should be taken into account. A smaller amount of iron deposition in the basal ganglia in female p.C282Y homozygotes has also been reported [43], which may be attributed to the prevention of iron accumulation from reaching a certain threshold due to menstruation, at least up to a certain age. Another reason is that estrogens play an antioxidant role moderating the damaging effect of iron and thus make women more resilient to its accumulation [44].

Most cases with HH and movement disorders carried the homozygous C282Y pathogenic variant, and less commonly were compound heterozygotes for C282Y and H63D variants. Although homozygosity for the H63D mutation results in elevated intracellular iron and serum markers (ferritin, transferrin saturation), it rarely leads to clinical iron overload [45,46,47], unless combined with other mutations [48]. As shown in Table 1, only two more cases with H63D homozygous mutation exhibiting movement disorders have been described, suggesting very low penetrance of neurological symptoms for the latter [12,13]. Except for the homozygous H63D mutation, our patient also carried the c. 340+4T>C polymorphism in the HFE gene. Although it is considered non-pathogenic according to ACMG criteria [20], this polymorphism, when associated with the H63D change, could serve as a genetic modifier increasing the pathogenicity of the mutation. Indeed, the c. 340+4T>C polymorphism leads to a twofold increase in iron levels in carriers compared to healthy subjects [49]. Consistent with this view is the identification of SNPs in “modifier” genes that may influence iron-related parameters or patients’ phenotypes in HH [50,51,52,53,54,55].

With regard to pathophysiology, iron deposition in the basal ganglia and cerebellum, which are key components of the cortico-basal ganglia-thalamic circuits, can lead to movement disorders [56,57]. In patients with HH and movement disorders as well as in carriers of C282Y or H63D variants without neurological and liver manifestations [21,58,59,60], neuroimaging may show iron deposition in the basal ganglia, i.e., caudate, globus pallidus, red nuclei, substantia nigra, putamen and dentate nuclei, detected as T2 hypointensity, T1 hyperintensity and low signal on susceptibility weighted imaging [9,10,11,43,59]. Moreover, a large cross-sectional study showed that patients with p.C282Y homozygosity, the most prominent genetic risk factor for HH, was associated with increased iron deposition in localized subcortical motor circuits, i.e., basal ganglia, thalamus, red nucleus, cerebellum and an increased risk for movement disorders in general and, in particular, essential tremor and Parkinson’s disease [43]. The susceptibility of the basal ganglia to iron deposition may result from selective neuronal uptake and abnormal vascular or axonal transport of iron along white matter tracts connecting these nuclei [61].

Among previous described cases with movement disorders and HH, there was significant variability in neuroimaging findings, with many cases exhibiting iron deposition in the basal ganglia or cerebellum [9,10,11,21,22,24,29], while other cases exhibited normal brain MRI [6,13,25,27]. Notably, many cases shared the same movement disorders, such as tremor or parkinsonism [6,9,10,21,24,27], independent of brain iron deposition. On the other hand, some of the patients with no evidence of iron on brain MRI, had deposition of iron in the liver, confirmed by a biopsy [6,27]. Iron is an essential element, which is particularly important for neural development, myelination and synthesis of neurotransmitters, i.a. monoamines [62]. Dopamine neurons in particular, rely on iron for dopamine synthesis [63] and mitochondrial function [64], and are particularly vulnerable to iron dysregulation [65]. Excess iron leads to oxidative stress-mediated neurotoxicity or neurodegeneration [39]. Moreover, glial cells and especially microglia, mediate central disruption of iron homeostasis and are drivers of neuronal iron-dependent death [65,66]. Central iron regulation seems to have distinct mechanisms compared to the periphery [65]. Serum ferritin, for example, has a poor correlation with brain iron deposition [21,65], which could explain normal ferritin levels in few cases with HH and restless legs syndrome [36].

In the case of iron dysregulation, such as in the case of HH with neurological involvement and other neurodegenerative diseases [39], it seems that there is a rather regional iron misdistribution than global iron overload, in which individuals exhibit greater and lower iron levels in different brain regions simultaneously [65]. This could be a possible explanation, for the reduced iron level in the basal ganglia, measured by R2’ relaxometry, in two cases with HH and restless legs syndrome, despite systemic iron overload [28]. Moreover, it is possible that conventional MRI cannot depict the iron dysregulation that occurs in cellular and biochemical levels in many instances. The absence of abnormalities on conventional MRI is analogue to other metal accumulation disorders such as Wilson’s disease, where advanced imaging techniques such as diffusion tensor imaging (DTI) and volumetric studies can show brain microstructural abnormalities in the basal ganglia (and other areas), despite normal brain MRI, i.e., not showing copper accumulation [67,68]. Whether other factors such as genetic background, especially genes encoding proteins involved in iron metabolic pathways (such as the modifier DNA polymorphism detected in the present case), dietary preferences, altered function of blood–brain barrier, which normally protects the brain from iron overload, or glial microenvironment, affect central iron accumulation is yet not fully elucidated.

Regarding response to treatment, most movement disorders in HH showed a good response to symptomatic medication. In particular, parkinsonism was, in many cases, levodopa-responsive [6,9,24,25]. In addition, there has been one case of drug-resistant parkinsonism responding to deep brain stimulation [10]. One case of hand dystonia had an excellent response to botulinum toxin [21]. Phlebotomy had rather inconclusive outcomes with regard to movement disorders. Whether phlebotomy affects iron deposition on MRI was, in most cases, not reported; in two cases with no clinical effect, there was no change in iron deposition on MRI either [10], or there was even progression of deposition [22]. It seems that phlebotomy may have a positive effect on the movement disorder, when the latter is not long-standing [8,11,24,27].

In the present case, pallidal iron deposition contralaterally to hemidystonia, supports a significant link between HH and movement disorder. Indeed, a greater iron accumulation (lower signal in SWI) was detected focally in the left pallidum and less so in the right pallidum, consistent with the asymmetrical presentation of dystonia. A somatotopic organization of the limbs in the pallidum has been reported [69,70]. The rarity of this case lies in the rather acute or subacute nature of its presentation with a movement disorder, its non-progressive nature over almost 40 years, and the remission of some of the initial symptoms such as bulbar dysfunction. As noted above, while the H63D variance is substantially higher than the C282Y mutation in the general population, its neurological manifestations are exceedingly rare [14]. While the reasons for this are unclear, the harboring of the c. 340+4T>C polymorphism in the HFE gene of our patient provides an important clue to the mechanisms underlying H63D pathogenicity. Regarding the subacute onset of symptoms, the possible role of an intervening exogenous factor, such as viral infection, should be considered. This could have acted as an epigenetic trigger factor for the manifestation of this genetically determined condition [25]. The constitutional symptoms of the patient at the beginning of his disease, taken together with the increased ceruloplasmin levels, which acts as an acute phase protein, could point to this direction. The same applies for the development of oculogyric crisis in the course of the disease. While the overwhelming majority of oculogyric crises are caused by antipsychotic drugs, this symptom has also been linked to encephalitis [71,72,73]. However, it should be acknowledged that reduction in or withdrawal of anticholinergic medication, as in the present case, may have contributed to the emergence of oculogyric crisis [74]. Similarly, early neurological deterioration has been reported in other metal accumulation disorders, such as Wilson’s disease [75]. This appears to be particularly true for patients with neuro-Wilson’s phenotype, in which systemic manifestations of copper overload may be lacking [75]. Regarding the lack of progression, several factors need to be considered. These may include the genotype of our patient in conjunction with environmental and dietary factors that can potentially modify gene expression and clinical presentation, which are also thought to be operational in Wilson’s disease [76]. It can also be hypothesized that the non-progressive nature of the disease in our patient could relate to the absence of other exogenous insult(s) as the one that precipitated disease onset during puberty.

## 5. Conclusions

This paper describes a rare case of non-progressive isolated dystonia with subacute presentation and contralateral pallidal iron deposition, found to have a homozygous H63D mutation. Hemochromatosis can be clinically asymptomatic, rendering its association with movement disorders likely underdiagnosed. Simple blood analysis, including serum iron, ferritin, and transferrin saturation levels, should be investigated in patients with a movement disorder of unknown etiology. Conversely, HH should be considered in the differential diagnosis of movement disorders when iron deposition is detected on neuroimaging. Additionally, monitoring brain iron with quantitative brain iron MRI sequences in HH patients could help identify individuals susceptible to neurological symptoms early and potentially guide treatment modifications

In any case, patients with movement disorders should undergo a thorough evaluation, excluding other causes, before attributing their movement disorder to hemochromatosis. Future studies should investigate the relationship between specific HFE gene variants and the phenotype of movement disorders. The advent of next-generation sequencing will help identify non-HFE mutations that could act synergistically as genetic modifiers influencing variable clinical expression of HFE mutations, for example, genes affecting iron regulation or the blood–brain barrier [5,65]. A better understanding of genotype-phenotype correlations would facilitate the early diagnosis of this inherited metabolic disorder, ultimately leading to timely interventions, mitigation of symptoms, and normal life expectancy.

## Data Availability

Data is contained within the article; further inquiries can be directed to the corresponding author.

## Supplementary Materials

The following supporting information can be downloaded at: https://www.mdpi.com/article/10.3390/diagnostics15172275/s1, Video S1: hemochr dystonia video.
